# Mechanisms of damage and therapies for cardiac amyloidosis: a role for inflammation?

**DOI:** 10.1007/s00392-024-02522-2

**Published:** 2024-08-21

**Authors:** Ilaria Anna Bellofatto, Panagiota Efstathia Nikolaou, Ioanna Andreadou, Marco Canepa, Federico Carbone, Alessandra Ghigo, Gerd Heusch, Petra Kleinbongard, Christoph Maack, Bruno K. Podesser, Kimon Stamatelopoulos, Konstantinos Stellos, Gemma Vilahur, Fabrizio Montecucco, Luca Liberale

**Affiliations:** 1 Department of Internal Medicine, University of Genoa, 6 Viale Benedetto XV, 16132 Genoa, Italy; 2 Laboratory of Pharmacology, Faculty of Pharmacy, National and Kapodistrian University of Athens, Panepistimiopolis, Zografou, 15771 Athens, Greece; 3 Cardiology Unit, Ospedale Policlinico San Martino IRCCS, Genoa, Italy; 4 IRCCS Ospedale Policlinico San Martino Genoa–Italian Cardiovascular Network, L.Go R. Benzi 10, 16132 Genoa, Italy; 5 Department of Molecular Biotechnology and Health Sciences, Molecular Biotechnology Center “Guido Tarone”, University of Torino, Turin, Italy; 6 Institute for Pathophysiology, West German Heart and Vascular Center, University of Duisburg-Essen, Essen, Germany; 7 Department of Translational Research, Comprehensive Heart Failure Center (CHFC), and Medical Clinic I, University Clinic Würzburg, Würzburg, Germany; 8 Ludwig Boltzmann Institute for Cardiovascular Research at the Center for Biomedical Research and Translational Surgery, Medical University of Vienna, Vienna, Austria; 9 Angiology and Endothelial Pathophysiology Unit, Department of Clinical Therapeutics, Medical School, National and Kapodistrian University of Athens, Athens, Greece; 10 Department of Cardiovascular Research, European Center for Angioscience, Medical Faculty Mannheim, Heidelberg University, Mannheim, Germany; 11 Research Institute, Hospital de La Santa Creu I Sant Pau, IIB-Sant Pau, C/Sant Antoni Mª Claret 167, 08025 Barcelona, Spain; 12 CiberCV, Institute Carlos III, Madrid, Spain

**Keywords:** Inflammation,, Amyloidosis, Cardiac amyloidosis, Light chain amyloidosis, Transthyretin amyloidosis

## Abstract

The term cardiac amyloidosis (CA) refers to the accumulation of extracellular amyloid deposits in the heart because of different conditions often affecting multiple organs including brain, kidney and liver. Notably, cardiac involvement significantly impacts prognosis of amyloidosis, with cardiac biomarkers playing a pivotal role in prognostic stratification. Therapeutic management poses a challenge due to limited response to conventional heart failure therapies, necessitating targeted approaches aimed at preventing, halting or reversing amyloid deposition. Mechanisms underlying organ damage in CA are multifactorial, involving proteotoxicity, oxidative stress, and mechanical interference. While the role of inflammation in CA remains incompletely understood, emerging evidence suggests its potential contribution to disease progression as well as its utility as a therapeutic target. This review reports on the cardiac involvement in systemic amyloidosis, its prognostic role and how to assess it. Current and emerging therapies will be critically discussed underscoring the need for further efforts aiming at elucidating CA pathophysiology. The emerging evidence suggesting the contribution of inflammation to disease progression and its prognostic role will also be reviewed possibly offering insights into novel therapeutic avenues for CA.

## Introduction

**Fig. 1:**
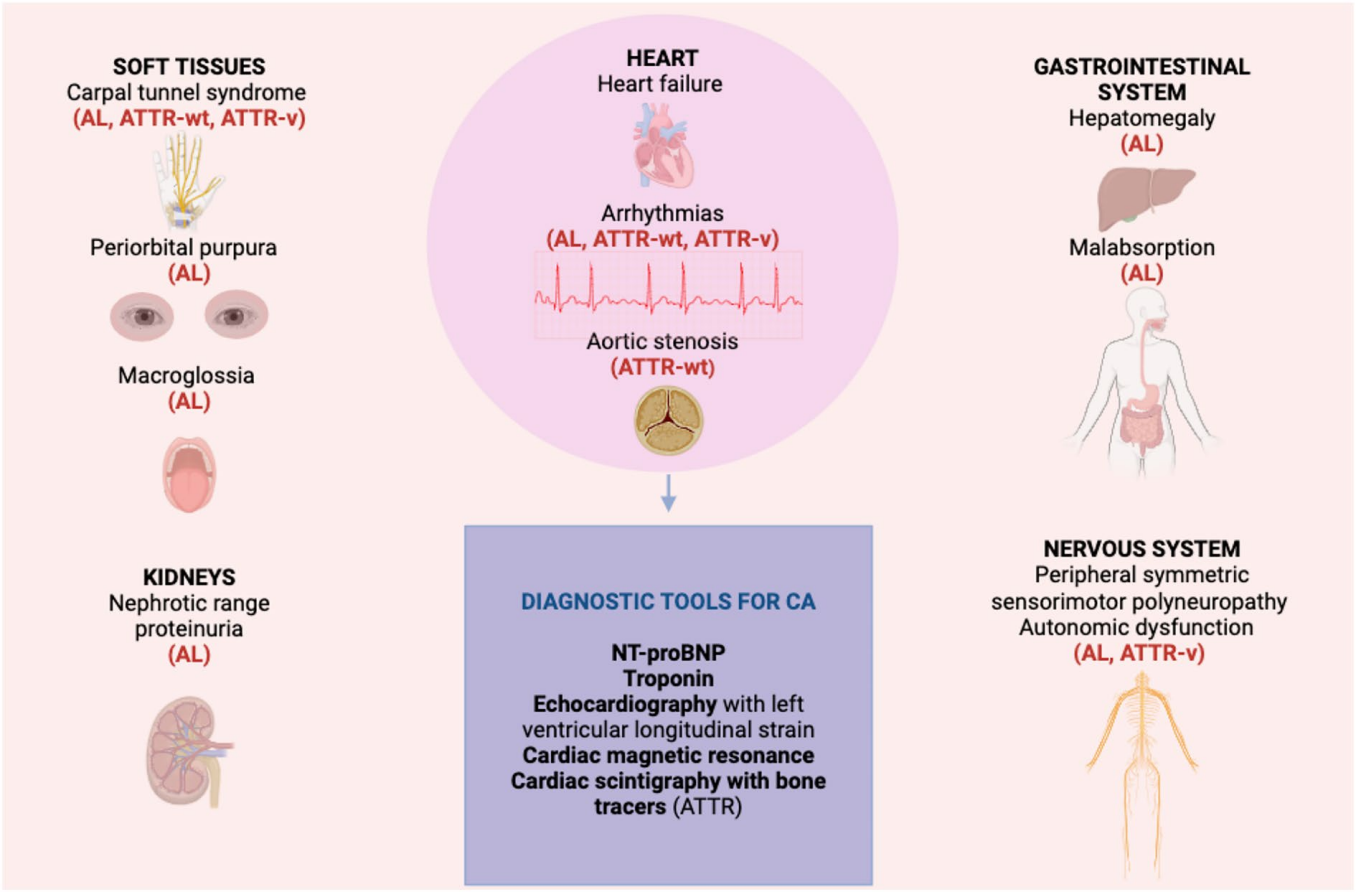
Organ involvement in systemic amyloidosis and diagnostic tools for detecting cardiac amyloidosis. *AL* Light chain amyloidosis, *ATTR-v* variant Transthyretin amyloidosis, *ATTR-wt* wild-type Transthyretin amyloidosis, *NT-proBNP* N-terminal pro brain natriuretic peptide. *Source*: Created with BioRender.com

The term amyloidosis encompasses a group of heterogenous conditions characterized by the deposition of amyloid fibrils in the extracellular matrix of tissues and organs (Fig. 1) [1]. More than 40 precursor proteins are known to misfold and self-assemble as amyloids with highly ordered cross beta-sheet conformation identified by electron microscopy and a characteristic apple-green birefringence under polarized light when stained with Congo red [2, 3]. Among those, cardiac amyloidosis (CA), defined as the accumulation of amyloid fibrils in the heart, is predominantly associated with light chain (AL) or transthyretin (ATTR) amyloidosis.

Both AL and ATTR are systemic amyloidosis types since the anatomic site of the precursor protein synthesis is remote from the organs with amyloid deposition [4]. AL amyloidosis is classified as a plasma cell dyscrasia with relatively low levels of clonal plasma cells in the bone marrow which cause the overproduction of immunoglobulin light chains of either the kappa or lambda family. The clonal light chain undergoes misfolding and aggregation into oligomers. These oligomers then build up in fibrils, which are then deposited in the extracellular space of different tissues [2, 5]. AL amyloidosis primarily affects the heart and kidneys, although it can potentially involve every organ of the body, except for the central nervous system; its clinical representation being dependent on the type and the extent of organ deposition [2, 6]. On the other hand, ATTR is characterized by transthyretin (TTR) deposits and comprises two subtypes named ATTR variant (ATTR-v) and ATTR wild type (ATTR-wt) [7]. TTR is a protein mainly synthesised by the liver and functions as a plasma carrier for thyroxin and retinol. While it usually organises into tetramers, protein instability promoted by aging or pathogenic variants in the TTR gene can cause its dissociation into monomers, which consequently misfold and aggregate into fibrils accumulating in tissues [7]. Dozens of point mutations in the TTR gene have been identified to cause ATTR-v, determining a variable spectrum of manifestations but predominantly involving the heart and nervous system and including small fibre neuropathy and autonomic dysfunction [2]. ATTR-wt, previously referred to as “senile systemic amyloidosis”, has a strong male and cardiac predominance while peripheral nerves and soft tissues may also be involved [8]. Specifically, bilateral carpal tunnel syndrome, spinal stenosis and biceps tendon rupture are the common extra-cardiac manifestations [8].

This review reports on the cardiac involvement in systemic amyloidosis, its prognostic role and how to assess it. Current and emerging therapies will be critically discussed underscoring the need for further efforts aiming at elucidating CA pathophysiology. While several mechanisms of damage have been recognized, the role of inflammation remains incompletely understood. Here we also review the emerging evidence suggesting its contribution to disease progression and its prognostic role and possibly offering insights into novel therapeutic avenues for CA.

## Cardiac involvement as a prognostic factor

Cardiac involvement is a common manifestation of AL, ATTR-wt and ATTR-v, and it is associated with reduced survival regardless of the amyloid type [9, 10]. In both AL- and ATTR-CA, extracellular amyloid deposits contribute to progressive ventricular wall thickening and stiffness, resulting in diastolic dysfunction and eventually heart failure [11, 12]. The typical phenotype is heart failure with preserved ejection fraction (HFpEF), although the progressive damage can eventually impair systolic function as well [11, 13–16]. CA may also manifest with conduction abnormalities and arrhythmias such as atrial fibrillation, arising from atrial infiltration and remodelling secondary to diastolic left ventricular dysfunction with elevated filling pressures [17–21]. Amyloid fibrils can infiltrate any cardiovascular structure and potentially contribute also to the onset or progression of aortic valve stenosis [22–25]. Atrioventricular valve thickness is more prevalent in ATTR while hemodynamic impairment is more prominent in AL [26]. Additionally, coronary microvascular dysfunction contributes to the CA phenotype resulting from capillary rarefaction, extrinsic compression of the microvasculature and autonomic and endothelial dysfunction and justifying the rise in troponin levels despite the absence of significant epicardial coronary lesions [27–31]. ATTR-CA shows important sex-related differences in term of epidemiology, clinical presentation and diagnosis, as well as treatment and prognosis. This disease is indeed less prevalent in women with an increased postmenopausal incidence; left ventricular ejection fraction is usually preserved with increased diastolic and right ventricular dysfunction. Also, some reports suggest reduced survival although this is to be confirmed in larger registers [32].

Cardiac amyloid burden within each subtype correlates with mortality [33, 34]. The extent of cardiac involvement (typically estimated by ventricular wall thickness using echocardiography or more accurately by extracellular volume and T1 mapping sequence using CMR) shows considerable variation among different amyloidosis types [35]. Specifically, ATTR-wt displays the highest average wall thickness values, 3 to 4 mm thicker than those observed in AL or ATTR-v subtypes. Yet, patients with AL-CA showed worse outcomes than those with ATTR-CA, at least partly attributed to higher cardiotoxicity of free light chains and their oligomers, prompting various study groups to include blood-borne cardiac biomarkers of myocardial injury and strain in their prognostic staging systems [36–39]. In AL, N-terminal pro-brain natriuretic peptide (NT-proBNP), cardiac troponin I and T (cTnI, cTnT) and the difference between involved and uninvolved free light chains (dFLC) are the main determinants in patient stratification and prognosis even in those without cardiac involvement [38]. Similarly, in ATTR, NT-proBNP has a significant prognostic value and is incorporated in both available staging systems along with cTnT and estimated glomerular filtration rate [36, 37].

Cardiac magnetic resonance (CMR) has shed light on the extent of cardiac involvement and its correlation with prognosis and provided insights into the response to infiltration in terms of myocyte hypertrophy or loss [40, 41]. In both AL- and ATTR-CA, increased extracellular volume is a surrogate marker of the interstitial space expansion due to extracellular amyloid deposits and is associated with adverse patient outcomes [11, 42]. In ATTR, myocyte volume is increased to an extent where some authors have proposed to include TTR gene variants in the hypertrophic cardiomyopathy genetic screening [43–45]. In contrast, in AL, myocyte volume is either unchanged or minimally increased and hypertrophy is offset by the concomitant cellular loss due to cardiotoxicity [41, 46]. CMR tissue characterisation expands our understanding of AL and ATTR cardiomyopathy allowing incorporation of CMR markers in classifications and prognostic systems, which are currently being developed [47–49].

Timely diagnosis of CA is imperative to achieve higher response rates to treatment, particularly for AL amyloidosis, which carries a time-sensitive grim prognosis largely affected by initiation of treatment [38, 50]. Early diagnosis and subtyping requires a high degree of suspicion, an experienced multi-disciplinary team in addition to cardiologists, the availability of specialized diagnostic tools and expertise to utilize and interpret their results, [51, 52] highlighting the need for centres of excellence in amyloidosis. The workflow for diagnosis and phenotyping is summarized elsewhere [53–55]. Diagnostic tools for detecting cardiac involvement in AL or ATTR amyloidosis are summarised in Fig. 1. Besides elevated troponin levels, NT-proBNP levels are usually disproportionately raised relative to the patient’s hemodynamic status [56]. Typical echocardiographic findings include increased thickness of ventricular free walls, septum and valves, with a bright echo texture of the myocardium known as “sparkling” appearance. Characteristically, there is an abnormal longitudinal strain pattern with sparing of the left ventricular apex [5]. CMR normally shows an increased wall mass with a diffuse non-ischemic pattern of delayed gadolinium uptake [56]. Bone scintigraphy represents a non-invasive option for diagnosing ATTR amyloidosis with a very high sensitivity and specificity [57]. Recent evidence suggests a possible role for positron emission tomography (PET)/CT scans with tracers holding amyloid-specific but not subtype-specific affinity [58]. Its spatial resolution, together with the possibility to quantitative analyse the tracer uptake may find a role in estimating amyloid burden and assessing disease trajectories in response to therapy. Different explorative studies have investigated such potential applications for both ATTR and AL amyloidosis; a recent meta-analysis reported a sensitivity of 95% and a specificity of 98% for the detection of CA by PET [59].

## Mechanisms underlying cardiac damage

The mechanisms underlying organ damage in CA are multifactorial, entailing both proteotoxicity as well as the mechanical consequences of amyloid fibrils in tissue structure [60]. Organ dysfunction observed in CA cannot be solely attributed to mechanical interference with tissue architecture, and the mechanisms of damage at the cellular level are intricate and mostly undefined [61]. Plenty of experimental and clinical evidence indicate a discrepancy between amyloid burden and the grade of cardiac impairment and survival in cases of AL cardiomyopathy [26, 62, 63] Soluble pre-fibrillar amyloidogenic light chains (LCs) directly damage cardiac cells, as observed in experimental systems using animal models to reproduce LCs mediated cardiotoxicity [64–66]. Upon the first observation that LCs from urine of patients with AL-CA can cause diastolic dysfunction in isolated murine hearts [63], it was further demonstrated that they increase oxidative stress in isolated rat ventricular cardiomyocytes and result in direct impairment of cardiomyocyte calcium handling and contractile function [67]. The damage is determined by amyloidogenic and cardiotropic LCs, leading to cellular dysfunction in terms of reduced viability, increased reactive oxygen species (ROS) production and mitochondrial damage [68]. Stress-activated kinases and ROS production have been proved to be downstream elements of the cardiomyocyte response to AL-LCs [66, 67, 69]. Moreover, the increased levels of ROS appear to stem from a disrupted clearance process of damaged cardiac cell mitochondria, secondary to AL-LC-induced lysosomal dysfunction with a dysregulated autophagy [70]. Changes in cellular proteome have been documented in colonies of human cardiac fibroblasts (hCFs) exposed to amyloidogenic cardiotropic LCs, leading to alterations in cell physiology with increased apoptosis [62].

Existing data from in vitro experiments suggest that circulating TTR amyloid fibrils or prefibrillar proteins could trigger inflammation, generate ROS, induce apoptosis, and stimulate autophagy before their deposition, exerting a toxic effect on cardiomyocytes [60, 71, 72]. The ex-vivo interaction of pre-fibrillar V122I-TTR variant and cardiomyocytes in tissue culture systems decreased cell viability by affecting cell surface characteristics or modifying protein and ion transport [73]. Different TTR intermediates of fibrillogenesis have been isolated in vitro, with smaller ones (< 100 kDa) capable of inducing cardiac cell damage through apoptosis [72]. In senile amyloidosis, oxidised forms of the TTR protein are particularly prone to aggregation and fibril formation, exerting a toxic effect on cardiomyocytes with a dose-dependent gradient. This phenomenon is particularly noteworthy in the context of aging, which is characterized by an abundance of oxidised proteins in both plasma and tissues [74, 75]. This toxic effect can lead to tissue damage before TTR deposits are detectable, as shown in biopsies of asymptomatic carriers of mutant TTR. The same mechanism was observed in familial amyloid polyneuropathy, with neuronal loss resulting from a combination of degeneration due to displacement of peripheral nerve fibres secondary to amyloid infiltration and the neurotoxicity of pre-amyloidogenic forms of TTR [76]. Clinical evidence supporting cardiotoxicity also arises from data indicating that a chemotherapy-induced reduction of at least 50% in circulating LCs leads to a clinically significant decrease in N-terminal pro-natriuretic peptide type B (NT-proBNP) [77]. NT-proBNP, BNP and troponins consistently demonstrate efficacy for assessing improvements in cardiac function following the reduction of LCs levels [77]. In this context, cardiac biomarkers not only serve as prognostic indicators [36–39] but can also reflect the cardiac response to therapy [78]. Furthermore, the swift decrease in NT-proBNP observed during treatment does not necessarily reflect a reduction in cardiac wall thickness, emphasising the direct role played by amyloidogenic precursors in causing damage to cardiac cells [77].

Mechanistic insights for cardiotoxicity of amyloid have yet to be obtained from animal models. The in vitro LCs cardiotoxicity form is confirmed in simple but vertebrate animal models. Injections of LCs in zebrafish led to increased cardiac cell death, resulting in fatal effects with a median survival of 5 days post-injection [79]. A transgenic zebrafish model demonstrated LC-induced cardiotoxic effects related to apoptosis and autophagy but did not affect overall lifespan, potentially due to increased cardiac tissue regeneration [64]. In mouse models producing free LCs at levels even surpassing those found in patients, spontaneous amyloidogenesis does not occur, indicating a potential resistance of mice to AL amyloidogenesis. Seeding with ex vivo prepared amyloids lead to amyloid deposition but cardiac toxicity and lifespan changes are not observed. Understanding the factors limiting clinical representation of amyloidosis in mice could prove immensely valuable [80]. The creation of animal models has been challenging for ATTR as well. Initial attempts yielded mice with minimal amyloid deposits. Subsequent improvements, including increased gene copies of TTR V30M, led to observable amyloid deposits in various organs, yet none fully replicated human pathology [81]. Crossing transgenic mice expressing human TTR V30M with those lacking heat shock transcription factor (Hsf1) revealed earlier and more extensive TTR deposition, offering insights into pathogenesis and potential therapeutic avenues despite limitations in model optimization [82]. Transgenic mice expressing TTRvS^52P^ bear amyloid fibrils, predominantly in the heart and tongue upon seeding, however, to the best of our knowledge cardiac function, amyloid microenvironment and mechanism driving organ tropism have not been examined, probably due to the lack of the model availability [83]. Indeed, while spontaneous ATTR amyloidosis mimicking the human cardiac phenotype is reported in vervet monkeys bearing amyloidogenic human alleles, to date no large mammals experimental model is available [84].

## Inflammation in amyloidosis

Inflammation has recently gained wide interest in cardiology. Milestone studies have elucidated the intimate relation between vascular inflammation and plaque vulnerability in patients with coronary artery disease (CAD) [85–87]. Along the same line, inflammatory activation has been linked to disease progression in chronic heart failure and several experimental attempts have been made to target cellular and soluble mediators [86–88].

The presence of inflammatory markers around amyloid aggregates is a well-recognised feature in various forms of amyloidosis [89, 90]. For instance, in Alzheimer’s disease (AD), the progressive accumulation of amyloid-beta (A*β*) peptides stimulates an inflammatory response in the cerebral cortex, contributing to the pathogenesis of the disease [15]. Experimental evidence suggests TNF-*α* inhibitors as effective strategies to reduce inflammation-related damage and cognitive decline [91, 92]. Of much interest, active immunisation against A*β* proved effective in an experimental model of AD [93]. However, the phase 2 trial investigating the first generation of anti-A*β* vaccine had to be halted due to the occurrence of meningoencephalitis in a significant proportion of patients [94].

The evolution of the inflammatory changes during the course of AL and ATTR disease and its correlation to the cardiac involvement are poorly understood. The inflammatory cascades between the two types may share both common and distinct features. Since the basic mechanisms of amyloid formation are universal [95], common pathways of amyloid fibril deposition may include activation of immune cells, production of pro-inflammatory cytokines, and subsequent tissue damage triggering further inflammation. However, AL and ATTR arise as a result from different pathologies, i.e. in AL the clonal plasma cell dyscrasia that could impact on the inflammation initiation and propagation. The existing data linking inflammation with AL and ATTR are summarized below.

### Extracardiac inflammation in AL amyloidosis

To the best of our knowledge, preclinical experimentations and clinical studies evaluating the inflammatory responses are scarce in AL. One of the first observations suggesting a possible role in the disease, is the activation of complement cascades in nerve biopsies affected by AL and ATTR-v. Interestingly, the complement expression was observed also in the vessels baring amyloid deposits triggering the investigation of chronic endothelial inflammation or injury in amyloidosis [96]. The possible implication of complement is also demonstrated by the transcriptional shift of AC16 cardiomyocytes upon treatment with recombinant AL fibrils in the presence of adipose-derived mesenchymal stromal cells towards a more immune-related phenotype. The authors suggest that the upregulated transcripts upon fibril exposure include immune response related genes such as complement component 3 (C3) and interleukins [96]. We suggest that in AL the underlying monoclonal gammopathy impacts on systemic inflammation. The clonal plasma cells express inflammatory markers in other plasma cell dyscrasias. Multiple myeloma plasma cells depend on cytokines for their growth, for example interleukin − 1 and − 6, and concomitantly they produce cytokines at high levels including interleukin − 6 and − 8 which correlate with disease progression and prognosis. To the best of our knowledge, interleukin production has not been identified or studied in AL [97–99]. The possible effect of LCs on inflammation is also described in multiple myeloma-mediated proximal tubulopathy, in which occasional inflammatory cell infiltration and interstitial fibrosis are observed. In this context, activation of nuclear transcription factor κ-B and mitogen-activated protein kinases, resulting in the transcription and subsequent release of interleukins -6 and -8 and monocyte chemoattractant protein 1 (MCP-1) were observed [100].

### Extracardiac inflammation in ATTR amyloidosis

In vitro studies have demonstrated the interaction between TTR and receptors for advanced glycation end products (RAGE) in familial amyloidotic polyneuropathy (FAP), leading to the induction of the transcription factor NF-kB. This interaction could consequently contribute to the expression of macrophage-colony stimulating factor, interleukin-6, and other inflammation-associated molecules [101]. The presence of inflammatory mediators in FAP nerve biopsies has been confirmed in a later study, where a semi-quantitative analysis of immunohistochemical images showed the expression of TNF-*α* and interleukin 1 beta (IL-1*β*) [102]. However, despite the local expression of these cytokines in peripheral nerves, inflammatory infiltrates of lymphocytes or mononuclear phagocytes were not detected in FAP lesions [102]. The association between non-fibrillar deposit of TTR in peripheral nerves and induction of pro-inflammatory cytokines has then been confirmed in a transgenic mouse model carrying the human TTR V30M gene (the most common mutation found in TTR-v) [82]. Another report using an animal model of TTR deposition to study disease development, highlighted the increased levels of inflammation-related transcripts in both liver and heart of transgenic mice, strengthening the concept that inflammation might play a role in the systemic progression of the disease [103]. More recently, novel murine models have been generated reproducing pathological findings of FAP. Yet, the inflammatory response was either not observed, or not described [104, 105].

### Cardiac inflammation

With regards to inflammatory mechanisms underlying cardiac amyloidosis, the evidence in the literature remains scarce. Despite the limited studies specifically targeting the relationship between CA and inflammation, valuable insights can be drawn from broader studies where amyloidosis was among the subpopulations of interest. First insights come from a study examining macrophages as potential markers of progression in a group of non-inflammatory myocardial diseases [106]. The analysis of macrophage receptor markers (MacR) in endomyocardial biopsies revealed significantly higher levels in samples from patients with a worse disease course, spanning conditions such as cardiac amyloidosis, cardiac Fabry disease, mitochondrial cardiomyopathy and biventricular arrhythmogenic right ventricular cardiomyopathy [106]. Interpreting these findings in the context of CA, the overexpression of MacR may result from the proliferation of normally limited resident inflammatory cells in response to damage induced by amyloid fibrils acting as damage-associated molecular patterns (DAMPs), either directly or through myocardial injury [106]. It is well known that DAMPs can activate tissue macrophages and dendritic cells through receptors like the Toll-like receptors [107, 108]. Additionally, the ability of amyloid fibres to trigger an immunological response via innate immune receptors has been demonstrated in other amyloidosis forms, such as lysozyme amyloidosis [109]. Lysozyme amyloid fibres can stimulate the production of pro-inflammatory cytokines through the activation of Toll-like receptor 2 and the NLR pyrin domain containing 3 inflammasome. Interestingly, this capability appears to be directly related not to lysozyme itself, but rather to the cross-beta fibrillar structure, common to all types of amyloid fibres [109]. Insights for the implication of the complement cascade in ATTR-CA were provided by diagnostic proteomics. Mass spectrometry-based proteomics is the gold standard for the amyloidosis tissue typing. The proteome map of intramyocardial cardiac biopsies from patients with AL and ATTR was compared to the expected proteomic diversity of cardiac tissue in non-amyloid control samples. In the amyloid-specific proteomes, ATTR was characterized by representation of complement proteins while this effect was not observed in AL. However, complement activation could also be related to age-specific effects in the ATTR population [110]. More recently, intramyocardial lymphocytic infiltration was reported in about half of myocardial biopsies from patients with CA and carry significant prognostic implications [111]. Specifically, the study included data from 54 patients affected by both AL and ATTR amyloidosis, who had been screened negative for coronary artery disease and other possible causes of left ventricular hypertrophy [111]. Myocardial inflammation turned out to be a marker of poor prognosis in all patients, with those suffering from AL amyloidosis having the poorest outcome compared to the other groups. Moreover, lymphocytes expressing CD3 receptor and lymphocyte function associated antigen 1 appeared to be the best predictive markers for poorer prognosis. [111] A noteworthy finding from this study is the lack of clinical surrogates to identify inflammatory phenotypes of cardiac amyloidosis. In fact, neither systemic biomarkers (i.e. NT-proBNP and troponin T) nor echocardiographic features significantly differed between inflammatory versus non-inflammatory groups, despite being increased in all patients [111]. This observation holds particular significance, especially considering that advancements in diagnostic tools have reduced the necessity for cardiac biopsy in making a diagnosis of cardiac amyloidosis. There is therefore a need for non-invasive tests to identify the “inflammatory phenotype” of CA [112].

### Inflammatory biomarkers of CA

In addition to the well-established late-stage biomarkers of CA, such as troponin and NT-pro-BNP, a new set of indicators focused on upstream events has been identified for their potential to detect misfolding events and the accumulation of pre-amyloid oligomers [113]. Within this category, osteopontin has found utility in the assessment of CA [113]. Osteopontin is a phosphoglycoprotein expressed by inflammatory and non-inflammatory cells, and has recently gained interest as a prognostic marker of heart failure and atherosclerosis [114]. Circulating osteopontin was shown to correlate with a more advanced cardiac disease in AL amyloidosis [115]. Yet, the low specificity of osteopontin reduces its potential as a biomarker [116, 117]. Similarly, the level of osteoprotegerin has also been linked to the level of cardiac involvement, and higher levels are associated with worse outcome [118]. Like osteopontin, also osteoprotegerin has been implicated in the pathogenesis of heart failure as an independent cardiovascular risk factor [119].

Belonging to the transforming growth factor beta superfamily, growth differentiation factor-15 (GDF-15) showed potential roles in inflammation, regulation of apoptosis, angiogenesis, and cell growth as well as in many cardiac conditions [120]. Circulating levels of GDF-15 are increased in elderly patients with frailty or acute illness and associates with inflammatory cytokines including interleukin 6 and tumor necrosis factor-*α* [121]. In patients with AL, circulating GDF-15 adds prognostic information on survival independent of that provided by established cardiac and renal risk biomarkers [122]. Inflammation is tightly interconnected with endothelial dysfunction and both play an important roles in many cardiac disorders [123]. While the pathophysiological role of endothelial activation in CA is under investigation, preliminary results suggest circulating levels of von Willebrand factor (vWF) to be independently associated with poor prognosis in patients with AL. [124] Similarly, preliminary results suggest soluble suppression of tumorigenicity 2 (sST2), an IL-33 decoy receptor, to be a biomarker of survival in AL [125]. In consideration of the anti-fibrotic role of such cytokine, its decoy receptor is expected to facilitate cardiac remodelling. The deposition of amyloid fibrils potentially impacts the extracellular matrix balance, which is strictly regulated by matrix metalloproteinases (MMPs) and their tissue inhibitors (TIMPs). Preliminary results reported a specific increase of MMP2, MMP-9 and TIMPs in patients with AL-CA but not in patients with ATTR-CA [126, 127]. Specifically, MMP9 and TIMP1 correlated with the degree of diastolic dysfunction in patients with AL-CA [127]. Finally, increased interleukin-6 levels were observed in ATTR-CA and were associated with inferior outcomes, but did not provide improved patient stratification beyond the established risk factors [128]. These results led the authors to conclude that interleukin -6 is linked to the heart failure rather than the underlying pathophysiology of amyloidosis.

## Current and emerging therapies

**Fig. 2:**
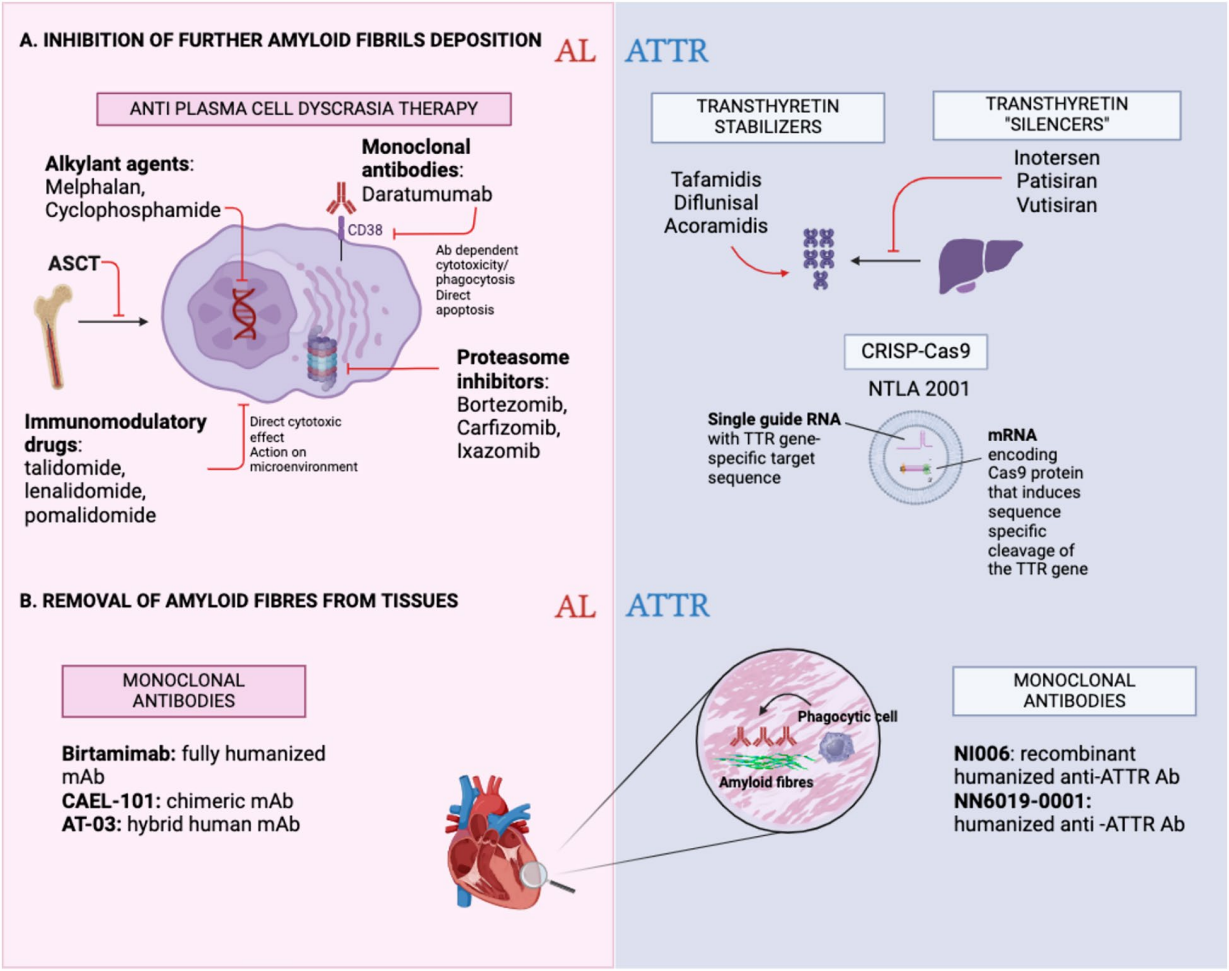
Therapeutic strategies for cardiac amyloidosis. In part A of the figure there is a list of agents directed at controlling further deposition of amyloid fibres in tissues. For AL amyloidosis, therapeutic schemes derive from those used for multiple myeloma. For ATTR amyloidosis, transthyretin “stabilizers” preserve the tetrameric structure of TTR, preventing its dissociation into amyloidogenic TTR monomers. Tafamidis is the only agent of this group currently approved for cardiac amyloidosis. Transthyretin “silencers” act by inhibiting TTR synthesis and expression. Inotersen is an antisense oligonucleotide complementary to the target mRNA, which blocks TTR synthesis. It is currently approved for patients with ATTR-v with stage 1 or 2 polyneuropathy. Patisiran and Vutisiran are small-interfering RNA able to degrade TTR mRNA and are approved for ATTR polyneuropathy. The CRISP-Cas9 system is based on the modification of a specific point of the target DNA using a guide RNA, aiming at correcting the mutation via its deletion or repair. NTLA2001 uses a lipid nanoparticle to deliver a mRNA encoding Cas9 protein and a single guide RNA that contains a TTR gene-specific target sequence. The result is a frameshift mutation in the TTR gene preventing the production of the TTR protein. In part B. we have listed the monoclonal antibodies created to remove amyloid fibrils from tissues. These agents are developed for the removal of amyloid by phagocytic immune cells. None of these has been approved yet. *ASCT* autologous stem cell transplant, *mAb* monoclonal antibody. *Source*: Created with BioRender.com

The identification of cardiac involvement and its extent is the primary determinant of prognosis and significantly influences therapeutic options of amyloidosis [9, 129, 130]. The therapeutic strategies are based on three different approaches: (1) elimination or reduction of the precursor protein production, (2) stabilization of the amyloidogenic protein or interference with the mechanisms of amyloidogenesis, and (3) amyloid removal or clearance from the organs involved. A detailed list of presently available therapies for both AL and ATTR is included in Fig. 2.

### AL amyloidosis

In AL amyloidosis, targeted therapies aim at eliminating the clonal cells which produce the light chain increasing survival [131]. They consist of autologous stem cell transplantation (ASCT) or anti-plasma cell chemotherapy/immunotherapy, which may themselves exhibit potential cardiovascular toxicity, especially in patients with multi-organ involvement [132]. The immunomodulatory anti-CD38 antibody daratumumab has been shown to benefit patients across all stages of cardiac involvement and remains the only agent specifically approved for AL amyloidosis [131, 133]. Previous studies have underscored the correlation between the severity of AL amyloid cardiomyopathy and outcome of ASCT, increasing mortality in these patients [33]. Therefore, nowadays, ASCT is an option only for a small number of patients, due to stringent eligibility criteria including age under 70 and preserved cardiac, hepatic, and renal function [134]. Novel AL treatments arise from the dynamic landscape of therapeutics in multiple myeloma, albeit with adaptations tailored to the unique characteristics of each condition. Despite therapeutic advances in AL, solely targeting the underlying clonal disease is inadequate, especially in patients with advanced organ damage or severe cardiac involvement who have limited therapeutic options [132, 135]. Survival is heavily dependent on early initiation of targeted treatment, which should be supported by general measures to manage heart failure [55].

### ATTR amyloidosis

The same concept as in AL amyloidosis applies to ATTR cardiomyopathy, where the current standard of care consists of preventing TTR fibril production either by “silencing” TTR synthesis and expression [136–139] or by stabilizing TTR tetramers to inhibit their dissociation into monomers [8, 140, 141]. TTR silencers such as Inotersen and Patisiran have been approved only for ATTR-v with stage 1 or 2 polyneuropathy and patients with ATTR polyneuropathy respectively. [142] Other targeted therapies to reduce the amyloid precursor protein production include CRISPR/Cas9 gene-editing therapy with NTLA-2001 which reduced TTR protein levels through targeted deletion of its gene [143]. Currently, the only approved treatment for ATTR-CA is the TTR stabilizer tafamidis, which has been included in ESC guidelines for the treatment of transthyretin amyloid cardiomyopathy, but only for patients with NYHA class I and II at baseline [8, 144]. Tafamidis improves all-cause mortality by delaying disease evolution versus placebo but it stabilizes rather than improves cardiac biomarkers and functional capacity [8]. A novel high-affinity TTR stabilizer, acoramidis, is the promising alternative to tafamidis for ATTR-CA by reducing mortality, cardiovascular-related hospitalizations with concomitant decrease in NT-ProBNP [140].

### Clearing amyloid depots

When the diagnosis is made early in the disease course, targeted therapies preventing or halting the cardiac deposition of amyloid fibrils can decelerate disease progression, reduce cardiovascular-related hospitalisations and improve functional status [2, 145, 146]. Despite recent evolutions, the pharmacotherapy for advanced cardiac amyloid infiltration and dysfunction is limited. For this reason, novel agents aiming at clearing amyloid depots have been identified and are currently under investigation in various experimental phases for both AL and ATTR. These therapies are based on the inflammatory system as they aim to activate complement-mediated macrophage and giant cell phagocytosis of amyloid fibril deposits from organs [147–150]. In AL, birtamimab and anselamimab (CAEL-101) have been developed as targeted anti-amyloid therapeutics. Birtamimab failed to reduce all-cause mortality in newly diagnosed patients in the phase 3 VITAL randomized controlled trial (RCT) but revealed promising outcomes among patients with advanced cardiac disease, which will be further assessed in the ongoing double-blind, placebo-controlled phase 3 trial (NCT04973137) [151]. CAEL-101 was developed to target a cryptic epitope on misfolded immunoglobulin light chains and fibrils. In phase 1 trials, CAEL-101 showed reductions in biomarkers associated with cardiomyopathy and nephropathy. Two concurrent randomized placebo-controlled double-blind phase 3 trials are actively recruiting patients with advanced cardiac disease (NCT04512235 and NCT04504825) and are expected to further define the role of anti-amyloid therapy in AL-CA [152].

Attempts to amyloid removal in ATTR are currently at an early clinical stage after showing preclinical efficacy. In a recent phase 1 trial NI006, a recombinant human anti-ATTR antibody inducing phagocyte-mediated removal of amyloidosis fibres, showed a good safety profile and reduced tracer uptake on cardiac scintigraphy as well as extracellular volume on CMR [148]. Whether removing deposited fibrils translates into a reduction of ATTR-CA-related symptoms in patients remains to be fully determined despite promising preliminary evidence [153]. This is the goal of an ongoing phase 2 RCT (NCT05442047) employing NN6019-000, another anti-ATTR antibody. However, since these agents are still in the early stages of testing, the current standard of care remains limited to reducing additional amyloid deposition [134].

### Conventional heart failure therapy

In the search for the ideal treatment regimen, the therapeutic management of CA can be challenging since patients do not usually respond to conventional heart failure therapies and due to the lacking of specific RCTs [27]. The restrictive nature of CA limits the ability to increase preload, making the use of beta-blockers detrimental, as increasing heart rate is the only way to improve cardiac output [56]. Preliminary observational studies suggest that low dosages of the cardio-selective beta-blocker bisoprolol could serve as a therapeutic option in ATTR-CA with LVEF < 40% since it is associated with improved survival [154, 155]. Similarly, renin–angiotensin–aldosterone inhibitors may be poorly tolerated, particularly by patients with autonomic dysfunction, due to the risk of hypotension [27]. Also, certain anti-arrhythmic drugs should be used with caution additionally considering a theoretical risk of increased accumulation of amyloid fibrils for digoxin [156]. Clinical observations suggest that mineralocorticoid receptor antagonists (MRA) are safe and tolerable in CA [157]. They have been associated with a lower risk of mortality ATTR-CA and this benefit is attributed to their synergistic effect with loop diuretics and increased potassium reabsorption, yet RCTs are needed to establish their efficacy. Recent preliminary evidence suggests a potential role for sodium-glucose cotransporter 2 inhibitors (SGLT2i) in improving volume status and reducing diuretic resistance [158]. Yet, most trials on SGLT2i excluded patients with CA, and no specific RCT is available for this population to date.

Given the shortcomings of currently available therapeutic strategies, it appears necessary to increase efforts to understand the mechanisms underlying amyloidosis-related cardiac damage in order to identify new potential targets. It remains to be resolved whether or not new therapeutic approaches to ATTR amyloidosis are able to modulate inflammatory biomarkers and thus help to identify underlying mechanisms in these patients.

## Conclusions

Currently there is not enough evidence to establish whether inflammation serves as a marker of advanced disease or actively contributes to disease development in CA, making it a potential target for therapy. This is at least partially due to the fact that reliable animal models are lacking while the existing ones are not yet characterized in terms of cardiac tropism and the development of inflammation. Furthermore, the prospective clinical evaluation of CA patients could facilitate the identification of systemic or localized inflammation markers in AL and ATTR. In consideration of the need to reduce intramyocardial biopsies, the investigation on tissues obtained in a less invasive way such as soft tissue biopsy, skin, lip and fat aspirate might facilitate the characterization of mediators of inflammation in the amyloid microenvironment. Lastly, as CA shows sex-dependent features, some mechanisms (potentially also inflammation) may play different roles in men and women, as shown for HFpEF [159], with different responses to targeted therapies. However, when analysing the potential of anti-inflammatory therapies in CA, it is important to recognise that, unlike other CV diseases such as myocardial infarction, in CA we cannot identify a single event that triggers the acute inflammatory response and guides our anti-inflammatory approaches. On the contrary, the chronic and low-grade nature of the inflammatory response in CA requires a different approach, probably milder and for longer periods of time, potentially carrying a higher rate of side effects. Further research is therefore needed to deepen our understanding of the impact of myocardial inflammation in cardiac amyloidosis and the potential therapeutic avenues it may offer.
